# Clinical, Immunological, and Molecular Heterogeneity of 173 Patients With the Phenotype of Immune Dysregulation, Polyendocrinopathy, Enteropathy, X-Linked (IPEX) Syndrome

**DOI:** 10.3389/fimmu.2018.02411

**Published:** 2018-11-01

**Authors:** Eleonora Gambineri, Sara Ciullini Mannurita, David Hagin, Marina Vignoli, Stephanie Anover-Sombke, Stacey DeBoer, Gesmar R. S. Segundo, Eric J. Allenspach, Claudio Favre, Hans D. Ochs, Troy R. Torgerson

**Affiliations:** ^1^Department of NEUROFARBA, University of Florence, Florence, Italy; ^2^Oncology/Hematology Department, “Anna Meyer” Children's Hospital, Florence, Italy; ^3^Seattle Children's Research Institute, University of Washington, Seattle, WA, United States

**Keywords:** immune dysregulation, polyendocrinopathy, enteropathy, X-linked (IPEX), IPEX-like, FOXP3, regulatory T cells, immune regulatory genes

## Abstract

**Background:** Immune Dysregulation, Polyendocrinopathy, Enteropathy, X-linked (IPEX) Syndrome is a rare recessive disorder caused by mutations in the *FOXP3* gene. In addition, there has been an increasing number of patients with wild-type *FOXP3* gene and, in some cases, mutations in other immune regulatory genes.

**Objective:** To molecularly asses a cohort of 173 patients with the IPEX phenotype and to delineate the relationship between the clinical/immunologic phenotypes and the genotypes.

**Methods:** We reviewed the clinical presentation and laboratory characteristics of each patient and compared clinical and laboratory data of *FOXP3* mutation-positive (IPEX patients) with those from *FOXP3* mutation-negative patients (IPEX-like). A total of 173 affected patients underwent direct sequence analysis of the *FOXP3* gene while 85 IPEX-like patients with normal FOXP3 were investigated by a multiplex panel of “Primary Immune Deficiency (PID—related) genes.”

**Results:** Forty-four distinct *FOXP3* variants were identified in 88 IPEX patients, 9 of which were not previously reported. Among the 85 IPEX-like patients, 19 different disease-associated variants affecting 9 distinct genes were identified.

**Conclusions:** We provide a comprehensive analysis of the clinical features and molecular bases of IPEX and IPEX-like patients. Although we were not able to identify major distinctive clinical features to differentiate IPEX from IPEX-like syndromes, we propose a simple flow-chart to effectively evaluate such patients and to focus on the most likely molecular diagnosis. Given the large number of potential candidate genes and overlapping phenotypes, selecting a panel of PID-related genes will facilitate a molecular diagnosis.

## Introduction

The Immune dysregulation, Polyendocrinopathy, Enteropathy, X-linked (IPEX) syndrome is a rare hemizygous disorder that presents most commonly in early infancy, but may occur antenatally (1) or later in life (2). The syndrome is characterized by severe enteropathy, chronic dermatitis, early onset type I diabetes mellitus (T1DM), hypoparathyroidism, antibody-mediated cytopenias, and other autoimmune phenomena (2). Affected males typically die within the first or second year of life if not treated with immunosuppressive agents or cured by hematopoietic stem cell transplantation (HSCT). IPEX was first recognized clinically in 1982 by Powell et al. who described a large kindred of five generations with 21 affected males who had all died prematurely (2). Linkage analysis localized the genetic defect to the centromeric region of the X chromosome (Xp11.23–Xq13.3) near the gene for Wiskott-Aldrich Syndrome (3, 4). Subsequently, mutations in the *FOXP3* gene, which codes for a DNA-binding factor with homology to Forkhead family proteins, were found to be associated with the IPEX syndrome (5–8), following the discovery of a frameshift mutation in *Foxp3* observed in a naturally occurring mutant mouse (9). The *scurfy* mouse was described more than 55 years ago (10) as an X-linked disease that shares many phenotypic features with IPEX, including scaly skin, runting, diarrhea, progressive anemia, lymphadenopathy and hepatosplenomegaly (10, 11).

Subsequent studies have demonstrated that the clinical phenotype of the scurfy mouse is the result of immune dysregulation and loss of peripheral tolerance mechanisms due to uncontrolled proliferation of activated CD4+ effector cells (12, 13) and absence of CD4+CD25+ regulatory T cells (Treg) (14). Extensive multi-organ infiltration with lymphocytes and increased production of multiple cytokines result in death of affected mice by 3–4 weeks of age (10–13, 15). Foxp3 is a key modulator of Treg development as it orchestrates the transcriptional machinery to induce Treg-relevant genes, such as *Il2ra* (CD25) and *Ctla4* (16) and by acting as a transcriptional repressor (14, 17–19).

As in the mouse, FOXP3 is the key factor in human Treg development and competitiveness, and its function has been extensively investigated (20–23). In normal individuals 5–7% of CD4+ T cells have the characteristic properties of Tregs. In addition to the Treg population that develops in the thymus, referred to as natural Treg (nTreg) cells, there are Tregs that are “induced” in the periphery from naïve CD4+ effector cells, identified as iTregs (24). Helios, a member of the Ikaros family of transcription factors, is highly enriched in nTregs in contrast to iTregs, a fact that has been used as a marker of Tregs of thymic origin (25). FOXP3+ Treg cells express a number of cell surface molecules that play unique roles in the maintenance (CD25), trafficking (CD103), and function (CTLA4) of regulatory T cells (26, 27). Down-regulation of the IL-7 receptor α (CD127) is another useful marker to identify Treg cells (CD4+CD25highFOXP3highCD127low) (28). Tregs exert their suppressive effect either by direct cell-cell contact, allowing immunoregulatory receptors such as CTLA4 to engage and down regulate antigen-presenting cells including activated B cells via co-stimulatory molecules (CD80/CD86), or by secreting immunoregulatory cytokines such as IL-10 and TGFβ that suppress bystander effector cells without direct cell-cell contact (29, 30). Finally, CD25-expressing Treg cells may induce suppression by acting as “IL-2 sink” through the efficient absorption of free IL-2 by the overexpressed IL-2 receptor α (CD25), thus depriving effector cells of this crucial cytokine (31).

In addition to “classic IPEX” caused by mutations in *FOXP3*, there is an increasing group of patients with an IPEX-like phenotype in the presence of a wild-type *FOXP3* gene that are being investigated for single gene defects other than *FOXP3*. Examples are mutations in *CD25* or *STAT5b*, genes that code for molecules that are required for homeostasis, fitness and maintenance of Treg cells (32, 33) or heterozygous mutation in *CTLA4* resulting in CTLA4 haploinsufficiency, causing decreased capacity to mediate cell contact-dependent suppression (34). More recently, mutations in *LRBA* have been reported as a cause of IPEX-like clinical manifestations characterized by early-onset enteropathy and endocrinopathies (35). Moreover, mutations affecting genes that regulate the JAK-STAT signaling pathways activated by T and B cell receptors have recently been identified as causing immune dysregulation with an IPEX-like phenotype. Included in this group of gene defects are heterozygous activating mutations in the PI3 kinase subunit p110δ encoded by *PIK3CD* and the PI3K subunit p35 encoded by *PIK3R1*, and gain of function mutations (GOF) in *STAT1* and *STAT3* (36–40). Abnormal activation of innate immunity can also drive systemic autoimmune diseases such as heterozygous germline mutations in tumor necrosis factor-alpha-induced protein 3 (*TNFAIP3*), which encodes a negative regulator of the nuclear factor-κB (NF-κB) pathway (41).

In this collaborative study, involving Seattle Children's Research Institute (Seattle, USA) and Anna Meyer Children's Hospital, (Florence, Italy) we evaluated 173 affected patients (168 males, 5 females) from 143 unrelated families with a clinical phenotype suggestive of IPEX. Starting in 2001, we sequenced the *FOXP3* gene using genomic DNA from each qualifying patient. To more clearly delineate the relationship between the clinical and immunologic phenotypes and the genotype, we reviewed the clinical and laboratory presentation of each patient to determine if there are particular phenotypic features that are typical for IPEX and are more predictive of a *FOXP3* mutation.

Because half of the patients, whose phenotype suggested IPEX, did not have a mutation in *FOXP3*, we investigated if these two groups could be differentiated phenotypically by comparing clinical and laboratory data of patients with *FOXP3* mutations (IPEX, *n* = 88) to those lacking an identifiable *FOXP3* mutation (IPEX-like, *n* = 85).

## Materials and methods

### Patients

The study included 173 patients from 143 unrelated families referred to the two participating centers for mutational analysis of the *FOXP3* gene. A questionnaire was submitted to each referring physician to collect pertinent clinical, laboratory and demographic information on each patient at the time of submitting DNA for sequencing. The requested information included the patient's age at diagnosis and the major clinical manifestations, such as endocrinopathy, enteropathy, skin disease, other autoimmune phenomena (i.e., hemolytic anemia AHA, thrombocytopenia ITP, and neutropenia), and infections. We also requested information about the immune status, type of treatment, and response to therapy. Screening for genetic mutations in *FOXP3* was performed in patients who presented with early-onset and/or severe clinical manifestations resembling IPEX syndrome, in particular at least one of the major clinical findings (enteropathy, endocrinopathy, skin disease) associated with other autoimmune manifestations. Written informed consent for genetic investigations, approved by the local ethics committees, was obtained from each patient or a parent.

### Genomic DNA isolation, amplification, and FOXP3 gene sequence analysis

Genomic DNA was isolated from peripheral blood using the QIAamp DNA Blood Mini Kit (Qiagen, Germantown, MD and Hilden, Germany) according to the manufacturer's protocol. The entire coding sequence of *FOXP3*, including exon–intron junctions, was amplified using specific flanking intron primer pairs as previously reported (8) and standard PCR conditions. The upstream promoter region and a 348-bp fragment encompassing the first polyadenylation signal 880-bp downstream of the termination codon were also amplified using previously described primers (42). PCR products were sequenced using the BigDye Terminator Cycle Sequencing Kit (Applied Biosystems, Foster City, CA, USA) on an automated ABI PRISM 3130 Genetic Analyzer (Applied Biosystems) and compared with reported reference cDNA sequences, using guidelines provided by HGVS and Mutalyzer 2.0.26.

### Next generation sequencing (NGS) analysis of IPEX-like patients

A custom sequencing array was designed to target all coding exons and flanking sequences for a panel of 50 genes that are known to cause PIDs to investigate those patients without identifiable *FOXP3* mutations (Supplementary Table 1).

The panel was designed using a custom target in solution enrichment NimbleGen SeqCap EZ Choice Design (Roche Inc., Madison, WI, USA) and included genes associated with combined immunodeficiencies, immune dysregulation disorders, innate immunity defects, and autoinflammatory diseases. Genomic DNA (gDNA) was purified and resuspended in water using the DNA Clean & Concentrator-5 columns (Zymo Research Corporation, Irvine, CA, USA); 135 ng of purified gDNA was used for the fragmentation reaction. The libraries were prepared using the Kapa Hyperplus Kit according to the manufacturer's protocol (Kapa Biosystem), that provides for the ligation of different adapters in order to analyze up to 24 samples in a single sequencing run. The pool was hybridized to the SeqCap EZ Choice Library designed to capture the genes included in the panel. Sequencing was performed using the Illumina MiSeq platform according to the manufacturer's recommendations using MiSeq Reagent Kit v3 and a 150 bp paired-end chemistry (Illumina, San Diego, CA, USA).

Sequence reads were aligned to the NCBI37/hg19 reference genome using a pipeline based on BWA and Picard (https://broadinstitute.github.io/picard/). Variants were called using the GATK toolkit (43).

Variants were annotated with gene name and classified according to their position and effect (frameshift, truncating, splicing, coding non synonymous, coding synonymous, intronic) using the ANNOVAR tool (44). Variants localized in intronic regions outside the 30 bp exon flanking boundaries were excluded. Variants reported in the Exome Aggregation Consortium (ExAC) database (http://exac.broadinstitute.org/) and/or in the 1000 Genomes Project (http://www.1000genomes.org) and/or in the NHLBI Exome Sequencing Project (ESP, ESP6500 database, http://evs.gs.washington.edu/EVS), with a Minor Allele Frequency (MAF) >0.05 (5%) were dropped out. To further determine the variant's pathogenicity a systematic classification scheme was applied using a combination of prediction programs: SIFT (sift.bii.a-star.edu.sg), PolyPhen (http://genetics.bwh.harvard.edu/pph2/), and pMUT (http://mmb.pcb.ub.es/PMut/PMut.jsp) to distinguish potentially damaging variants from those predicted to have neutral effect.

Putative causative variants were analyzed by Sanger sequencing to confirm the NGS results and investigated in the parents of probands to check their inheritance status.

### Flow cytometry of FOXP3+CD4+CD25+ cells

Surface staining for CD4 and CD25 was performed by exposing the cells to the anti-CD4 (SK3, BD Biosciences, CA) and anti-CD25 (M-A251, BD Biosciences) monoclonal antibodies for 15 min in the dark using a 2% bovine serum albumin phosphate-buffered saline (PBS) mixture on freshly isolated PBMCs. Cells were washed in PBS and fixed for 30 min at 4°C using the eBioscience fixation/permeabilization kit, according to manufacturer's instructions. After washing in PBS and permeabilization (15 min at 4°C), the cells were incubated overnight at 4°C with anti-human FOXP3 antibody (259D, BioLegend, San Diego CA), washed, examined by flow cytometry (FASCanto II) and analyzed with FACSDiva Software (BD Biosciences). FOXP3+ positive signal was evaluated by comparing to the isotype signal. Gating strategy was performed by selecting lymphocytes based on physical parameters; CD4+ cells were selected from the lymphocyte population, and FOXP3+CD25+ cells from the CD4+ cell subpopulation. Values of CD25+FOXP3+Tregs are given as percentage within the CD4+ population.

### Statistical analysis

We used a 2-sided 2-sample test of proportions to determine if there is a difference in the occurrence of clinical manifestations and in the levels of serum immunoglobulins between the IPEX and IPEX-like groups at a significance level of ≤0.05. Differences in age onset of symptoms between IPEX and IPEX-like were evaluated by the Mann–Whitney test. Differences in CD25+FOXP3+ Treg expression in IPEX, IPEX-like, and control subjects were evaluated by the One-way ANOVA test. Kaplan Meier Survival analysis was performed to determine survival curves. A log rank test for equality of survivor functions was performed to determine differences in survival curves at a significance level of 0.05. All analyses were performed using Graphpad Prism 6 software.

## Results

We aimed to perform a detailed molecular, immunologic, and clinical evaluation of a cohort of patients presenting with phenotypic features suggestive of a diagnosis of IPEX syndrome. Based on the results of *FOXP3* gene sequencing, we classified patients into 2 groups: those with disease causing *FOXP3* mutations (IPEX kindreds) and those without identifiable *FOXP3* mutations (IPEX-like kindreds). To investigate whether the clinical and immunologic features of our patients correlated with this molecular categorization, we used a specifically designed questionnaire to be completed for each patient by the referring physician.

### Molecular analysis

#### IPEX cohort with FOXP3 mutations

**Figure 1:**
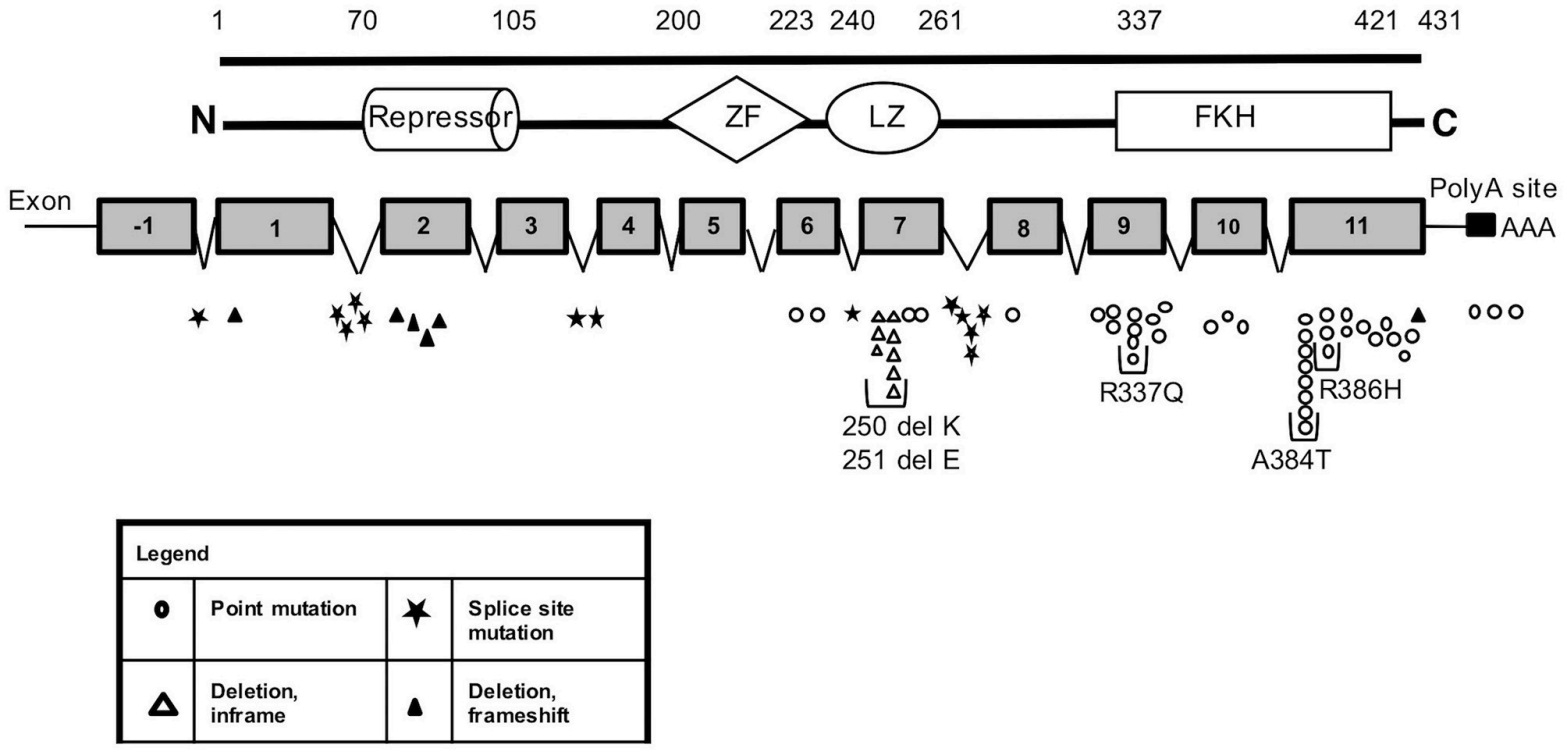
*FOXP3* mutations identified in our cohort of IPEX patients. Schematic representation of FOXP3 mRNA and protein showing the location and sizes of the predicted structural domains. Numbers along the top line indicate amino acid number (1–431). The position of the identified mutations along the mRNA is reported with different shape for each type of variation. Each symbol represents a single kindred. ZF, zinc finger; LZ, leucine zipper; FKH, forkhead box domain; *N*, amino terminus; C, carboxy terminus; PolyA, polyadenylation sequence.

**Figure 2:**
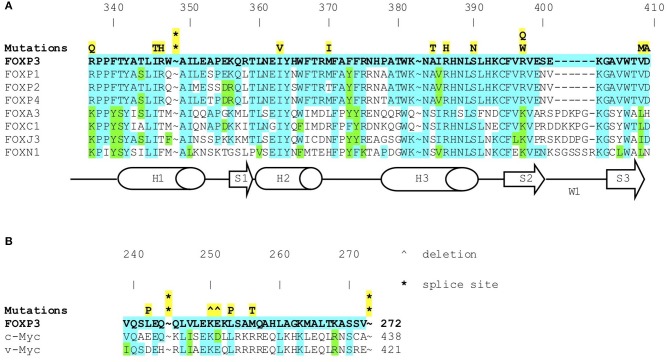
**(A)** *FOXP3* mutations in IPEX (symbols in yellow) in forkhead box domain. Amino acid sequence of FOXP3 protein aligned with other FOX proteins. Shows conserved residues (blue) and highly conservative residues (green). **(B)** *FOXP3* mutations in IPEX (symbols in yellow) in the leucine zipper domain. Amino acid sequence of FOXP3 protein aligned with c-myc and v-myc leucine zippers (known to be highly conserved). Shows highly conserved residues (blue) and highly conservative residues (green).

The molecular analysis of *FOXP3* carried out in 173 patients from 143 unrelated families enrolled identified *FOXP3* mutations in 88 affected patients (50.9%) from 66 (46.2%) unrelated families. Overall, 44 distinct sequence variants of *FOXP3* were identified, 9 of which were not previously reported (Table 1). The mutations were distributed throughout the *FOXP3* gene, although the majority were located within the functional domains, particularly the repressor N-terminal domain, the leucine zipper and the carboxy-terminal forkhead domain as shown in Figure 1. When aligning the forkhead domain and the leucine zipper region of the FOXP3 protein with members of other FOX protein family members, the mutations tended to occur at the most highly conserved residues (Figure 2).

**Table 1:** Mutations identified in the cohort of patients analyzed.

**Gene**	**Exon**	**cDNA**	**Protein**	**Mutation type**	**No. of families**	**References**
FOXP3^∧^1	−1	c.-6247_4859del	Large non-coding deletion	Splicing	1	(45)
FOXP3	1	c.29_47del	p.S10Lfs^*^46	Frameshift deletion	1	Unpublished
FOXP3	1+	c.210+1delG	Miss-spliced protein	Splicing	1	Unpublished
FOXP3	1+	c.210+1G>C	Miss-spliced protein	Splicing	1	(18)
FOXP3	1+	c.210+2del	Miss-spliced protein	Splicing	1	(46)
FOXP3	2	c.227del	p.L76Qfs^*^53	Frameshift deletion	1	(8, 47–49)
FOXP3	2	c.232_233del	p.M78Gfs^*^127	Frameshift deletion	1	Unpublished
FOXP3	2	c.239del	p.A80Dfs^*^49	Frameshift deletion	1	Unpublished
FOXP3	2	c.303_304del	p.F102Hfs^*^103	Frameshift deletion	1	(50)
FOXP3	3+	c.454+4A>G	Miss-spliced protein	Splicing	1	(51)
FOXP3	4-	c.455-1G>C	Miss-spliced protein	Splicing	1	Unpublished
FOXP3	6	c.694T>G	p.C232G	Missense	1	(52)
FOXP3	6	c.725T>C	p.L242P	Missense	1	(53, 54)
FOXP3	7–	c.736-2A>C	Miss-spliced protein	Splicing	1	(55)
FOXP3	7	c.748_750del	p. K250del	In Frame Deletion	3	(56–58)
FOXP3	7	c.751_753del	p. E 251del	In Frame Deletion	5	(5, 54, 59)
FOXP3	7	c.758T>C	p.L253P	Missense	1	(60)
FOXP3	7	c.767T>C	p.M256T	Missense	1	Unpublished
FOXP3	7+	c.816+2delT	Miss-spliced protein	Splicing	1	(61)
FOXP3	7+	c.816+3G>C	Miss-spliced protein	Splicing	1	Unpublished
FOXP3	7+	c.816+5G>A	Miss-spliced protein	Splicing	2	(53, 54)
FOXP3	7+	c.816+7G >C	Miss-spliced protein	Splicing	1	(62)
FOXP3	8	c.817G>T	p.A273S	Missense	1	(63)
FOXP3	9	c.967G >A	p.E323K	Missense	1	Unpublished
FOXP3	9	c.970T>C	p.F324L	Missense	1	(64, 65)
FOXP3	9	c.994A>G	p.K332E	Missense	1	Unpublished
FOXP3	9	c.1010G>A	p.R337Q	Missense	4	(66–68)
FOXP3	9	c.1037T>C	p.I346T	Missense	1	(61)
FOXP3	9	c.1040G>A	p.R347H	Missense	2	(61, 65)
FOXP3	10	c.1087A>G	p.I363V	Missense	1	(8, 56)
FOXP3	10	c.1099T>C	p.F367L	Missense	1	(69)
FOXP3	10	c.1110G>A	p.M370I	Missense	1	(70)
FOXP3	11	c.1150G>A	p.A384T	Missense	8	(7, 53, 71, 72)
FOXP3	11	c.1157G>A	p.R386H	Missense	3	(63)
FOXP3	11	c.1169G>A	p.S390N	Missense	1	(73)
FOXP3	11	c.1189C>T	p.R397W	Missense	2	(7, 56, 74)
FOXP3	11	c.1190G>A	p.R397Q	Missense	1	(63)
FOXP3	11	c.1222G>A	p.V408M	Missense	1	(66)
FOXP3	11	c.1226A>C	p.D409A	Missense	1	(75)
FOXP3	11	c.1227_1235del	p.D409_L411del	In Frame Deletion	1	(76)
FOXP3	11	c.1271G>A	p.C424Y	Missense	1	(56, 75)
FOXP3	11	c.1293_1294del	p.^*^432Text25	Frameshift deletion	1	(6)
FOXP3	Poly A	AAUAAA>AAUGAA		Unstable mRNA	2	(42)
FOXP3	Poly A	AAUAAA>AAUAAG		Unstable mRNA	1	(77)
IL2RA^∧^2	2	c.151T>G	p.C51G	Homozygous Missense	1	Paper submitted
IL2RA	2	c.452A>C	p.Q151P	Homozygous Missense	1	Unpublished
LRBA^∧^3	21	c.2496C>A	p.C832^*^	Homozygous Nonsense	1	Unpublished
LRBA	22	c.2617_2620del	p.L873Afs^*^13	Homozygous Frameshift deletion	1	Unpublished
LRBA	36	c.5680A>T	p.R1894^*^	Homozygous Nonsense	1	Unpublished
CTLA4^∧^4	2	c.436G>A	p.G146R	Heterozygous Missense	1	Unpublished
CTLA4	2	c.410C>T	p.P137L	Heterozygous Missense	1	(78)
STAT1^∧^5 GOF	12	c.1073T>G/T	p.L358W	Heterozygous Missense	1	(37)
STAT1 GOF	14	c.1154 C>T/C	p.T385M	Heterozygous Missense	1	(79)
STAT1 GOF	12	c.1053G>T/G	p.L351F	Heterozygous Missense	1	(80)
STAT1 GOF	11	c.983A>G/A	p.H328R	Heterozygous Missense	1	(81)
STAT3^∧^6 GOF	9	c.832C>T/C	p.R278C	Heterozygous Missense	1	Unpublished
STAT3 GOF	10	c.986T>G/T	p.M329R	Heterozygous Missense	1	Unpublished
STAT3 GOF	11	c.1079A>G/A	p.Y360C	Heterozygous Missense	1	Unpublished
STAT3 GOF	14	c. 1243 G>C/G	p.E415Q	Heterozygous Missense	1	Unpublished
STAT5b^∧^7	9	c.1680+1del		Splicing	1	(82)
TTC7A^∧^8		c.1817A>G/A; c.2086T>C	p.E606G, p.S696P	Compound Heterozygous Missense	1	(83)
TTC37^∧^9	33	c.859-3_859-7delTTTTT, c.3364delA	Miss-spliced protein, p.T1122Qfs^*^10	Compound Heterozygous Frameshift, Splicing	1	Unpublished
DOCK8^∧^10	27	c.3193del	p.S1065Afs^*^17	Homozygous Frameshift	1	(84)

*Nomenclature according to HGVS, using Mutalyzer 2.0.26 Name Checker and gene specific transcript variants. ^1^FOXP3, NM_014009.3; ^2^IL2RA, NM_000417.2; ^3^LRBA, NM_006726.4; ^4^CTLA4, NM_005214.4; ^5^STAT1, NM007315.3; ^6^STAT3, NM_139276.2; ^7^STAT5b, NM_012448.3; ^8^TTC7A, NM_001288951.1; ^9^TTC37, NM_014639; ^10^DOCK8, NM_203447.1; (PolyA, Polyadenylation site; fam, families)*.

Several residues with a high frequency of mutation rate were identified, with 5 mutations accounting for almost 35% of families (23/66) (Figure 1). These include three missense mutations in the forkhead domain, one occurring in exon 9 (c.1010G>A, p.R337Q) and two in exon 11 (c.1150G>A, p.A384T and c.1157G>A, p.R386H) and 2 single amino acid deletions in the leucine zipper region at residues 250 and 251 located in exon 7.

We identified 4 IPEX patients carrying deletion/frameshift mutations within exon 2 of the FOXP3 gene. In humans, two major alternatively spliced FOXP3 mRNA species have been described: the full-length (FL) isoform including all 11 coding exons, and a splice isoform lacking exon 2 (Exon 2^minus^). Under normal circumstances, the FL and exon 2^minus^ isoforms are the dominant species present, with each accounting for ~50% of the total FOXP3 mRNA in T cells (85–87). The two isoforms encode two proteins that can both be detected in T cells from normal individuals (85). The physiologic role of the short exon 2^minus^ isoform is unknown but their potential role in supporting Treg development has been investigated in a number of studies (88–92). The 4 patients reported here have deletion mutations in exon 2 that are predicted to cause a frameshift in the FL mRNA resulting in premature termination and absence of full-length mRNA that includes exon 2 while allowing expression of a normal exon 2^minus^ isoform of the protein. This was demonstrated by flow cytometry when we stained activated T cells from the patient with the c.239del mutation (and confirmed in the patient with the c.232_233del) using two different anti-FOXP3 antibodies, one that binds all FOXP3 isoforms and one that binds only within the sequence of the protein encoded by exon 2. The deletion mutations in exon 2 identified in the remaining 3 patients led to the expression of only the FOXP3 exon 2^minus^ isoform. This was demonstrated by Western blot when a genomic DNA fragment containing exons 1–7 from these patients was cloned into the pcDNA3-NV5 vector; in contrast, when a DNA fragment with wild type DNA fragment was cloned into the vector, equal amounts of both isotypes were expressed (Torgerson, Manuscript in preparation).

A second set of unique mutations identified in this large cohort of IPEX are mutations in the mRNA polyadenylation signal sequence of the *FOXP3* gene. The polyadenylation signal AAUAAA, located 798 bp downstream of the endogenous termination codon of the *FOXP*3 gene triggers polyadenylation of the FOXP3 mRNA, protecting it from degradation. A single nucleotide change (AAUAAA > AAUGAA) was identified in the first family described with IPEX syndrome (2, 42). Patients with this mutation were found to have only low-level expression of the *FOXP3* mRNA [(42) and data not shown], presumably due to increased degradation as a result of the absence of an appended poly-A tail. Since the discovery of this mutation, we have screened all patients with an IPEX phenotype and a normal *FOXP*3 coding sequence for mutations in the polyadenylation signal. As a consequence, we have identified 11 patients from 3 families with mutations in the *FOXP3* polyadenylation signal sequence, suggesting that up to 12% of patients with IPEX have mutations affecting the polyA site of *FOXP3*, a region that is largely neglected in usual sequencing approaches. Deletions of or mutations in the canonical polyA signal (AAUAAA), which triggers both cleavage and polyadenylation of pre-mRNA in the nucleus, prevent the addition of a long poly-adenosine tail to the 3′ end of the mRNA. This process is crucial for the transport of mature mRNA to the cytoplasm and for the stability of mRNA (6, 93).

A sequence variant (c.543C>T), although resulting in a synonymous amino acid substitution, was initially considered pathogenic since it affects the first base of exon 5, potentially resulting in a splicing defect. However, sequencing 200 chromosomes from a multiethnic population we found this alteration in 3.5% of this group of healthy individuals. To further evaluate the function of the splicing machinery in cells with the c.543C>T variant, we activated PBMCs from such patients to induce FOXP3 expression and then cloned and sequenced individual mRNAs. We found that in individuals with the c.543C>T alteration less than 5% of the transcripts misspliced around exons 4–5, compared to 0% in the controls. We do not believe this is enough dysfunction to result in the IPEX-like phenotype seen in these individuals, and therefore excluded them.

Another variation we reported previously (c.112G>T, p.A38S) (56), has been confirmed to be nonpathogenic. This alteration showed little difference from a wild-type FOXP3 vector in repression of a luciferase reporter containing an IL2 promoter. We had identified this variation in a family with 2 boys who presented with a severe IPEX-like phenotype including autoimmune enteropathy, eczema, diabetes, hypothyroidism, and nephrotic syndrome; both died prior to age 5. However, approximately 50% of asymptomatic men in this family were also found to have this alteration. For this reason, these boys have been included in the IPEX-like cohort.

#### IPEX-like cohort with wild type FOXP3

Overall, 85 patients (49.1%) with phenotypic features of IPEX from 77 unrelated families (53.8%) had no identifiable mutations in *FOXP3*. They were designated as IPEX-like and all Italian patients were evaluated for variants by a multiplex panel that included 50 genes involved in known PIDs. The Seattle patients were evaluated by Sanger sequencing of candidate genes (*STAT5b, STAT1, STAT3, IL2RA, CTLA4, LRBA, TTC7A, TTC37*). In this cohort of 85 affected individuals, 25 different disease-associated variants were identified in 21 IPEX-like patients (25%) affecting 9 distinct genes (Table 1). Seven of the nine genes had previously been associated with a phenotype of immune dysregulation including *LRBA, STAT1 (GOF), STAT3 (GOF), CTLA4, IL2RA, STAT5B*, and *DOCK8*. In particular, the “gain of function” mutations affecting the genes *STAT1* and *STAT3* account for 38% of the mutations identified in our IPEX-like cohort (8/21). All mutations identified in *LRBA* are novel and homozygous, two being nonsense mutations and one causing a frameshift; all are predicted to result in the absence of the protein or the formation of a truncated-dysfunctional protein. This is in agreement with most *LRBA* mutations reported to date (94).

All *STAT3* and *STAT1* mutations observed in our cohort are heterozygous missense variants. One of the four *STAT3* mutations located in the DNA binding domain, had been previously reported as a GOF mutation (78). Similarly, all *STAT1* mutations are located in the DNA binding domain and had been reported as GOF mutations by others (37, 79–81).

Mutations affecting *CTLA4* include two heterozygous missense variants, located in the extracellular domain of the protein: one had been reported to be associated with immune dysregulation (78, 95).

The *IL2RA* mutations identified include two novel homozygous missense variants, located in exons 2 and 4 that constitute the two “sushi” domains essential for IL2-IL2Rα interaction (96). These variants are associated with CD25 deficiency and will be reported in more detail separately.

The variants affecting *STAT5B*, a homozygous deletion resulting in a splicing defect, has been reported to be associated with Insulin-like growth factor (IGF) deficiency and immunologic defects (82).

Mutations in the dedicator of cytokinesis 8 (*DOCK8*) gene cause a combined immunodeficiency extending beyond recurrent infections to include atopy, autoimmunity and cancer (97, 98). DOCK8 deficiency has recently been identified in a patient with an IPEX-like disorder (99). Three brothers with IPEX-like phenotype were originally evaluated in our Centers and found to have wild type *FOXP3*. Subsequently, a homozygous single nucleotide deletion was identified in exon 27 of the *DOCK8* gene, resulting in frameshift and premature termination (84).

The two other genes we identified to be associated with IPEX-like Syndrome (*TTC37, TTC7A*) have not been implicated previously with immune dysregulation. The tetratricopeptide repeat domain 37 (*TTC37)* gene, if mutated, causes Trichohepatoenteric Syndrome (THES), a rare autosomal recessive disorder characterized by growth restriction, severe infantile diarrhea, trichorrhexis nodosa-like hair morphology, hepatopathy, facial dysmorphism, and immunodeficiency (100). The tetratricopeptide repeat domain 7A (*TTC7A*) gene is causative of hereditary multiple intestinal atresia (HMIA) (83), a rare cause of intestinal obstruction associated with a profound combined immune deficiency. Although these 2 disorders have not been directly associated with an IPEX-like phenotype, their clinical presentation can overlap. One of the compound heterozygous mutations in *TTC7A* identified in our patient has been previously described (83) while the two variants we observed in our patient with the *TTC37* were not.

The 21 detected variants associated with IPEX-like phenotype include 13 missense, 2 nonsense, 4 frameshift, and 2 splicing defects. Nine variants have previously been reported as disease causing in the Human Gene Mutation Database (HGMD; http://www.hgmd.cf.ac.uk/).

### Treg analysis by flow cytometry

**Figure 3:**
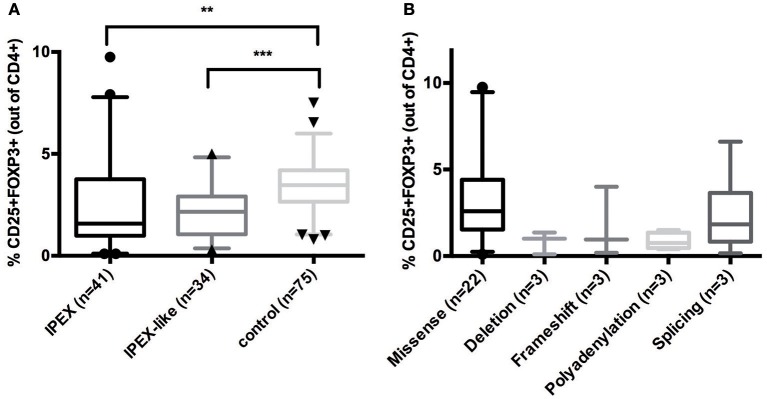
**(A)** CD25+FOXP3+ expression in IPEX, IPEX-like patients and control subjects. **(B)** IPEX patients differ according to the type of *FOXP3* mutations. Deletion = in frame deletion; frameshift = deletion with frameshift. Values are expressed as percentage of CD4 cells, median and 5–95 percentile are shown, circles, triangles, and cones represent singles patients out of 5–95 percentile (circles = IPEX patients, triangles = IPEX-like patients, cones = control subjects) (^**^p < 0.001; ^***^p < 0.0001).

To evaluate whether FOXP3 expression is normal or reduced in patients with IPEX and IPEX-like Syndromes, we compared 41 IPEX patients and 34 IPEX-like patients with control samples (*n* = 75) by flow cytometry. The percentage of FOXP3 expression by CD4^+^ lymphocytes in IPEX and IPEX-like patients was significantly lower compared to controls (Figure 3A). We also evaluated whether the percentage of FOXP3 expressing cells differed by the type of *FOXP3* mutations (Figure 3B) and found very low to almost no protein expression in patients with either in-frame or frameshift deletions, or polyadenylation site mutations, and variable expression in patients with missense and splicing mutations. Interestingly, two patients with missense mutations (p.E323K, p.S390N) had markedly elevated percentages of FOXP3 expressing CD4^+^ cells, which likely represent poor FOXP3 function, suggesting that the level of protein expression does not necessarily correlate with disease severity.

**Figure 4:**
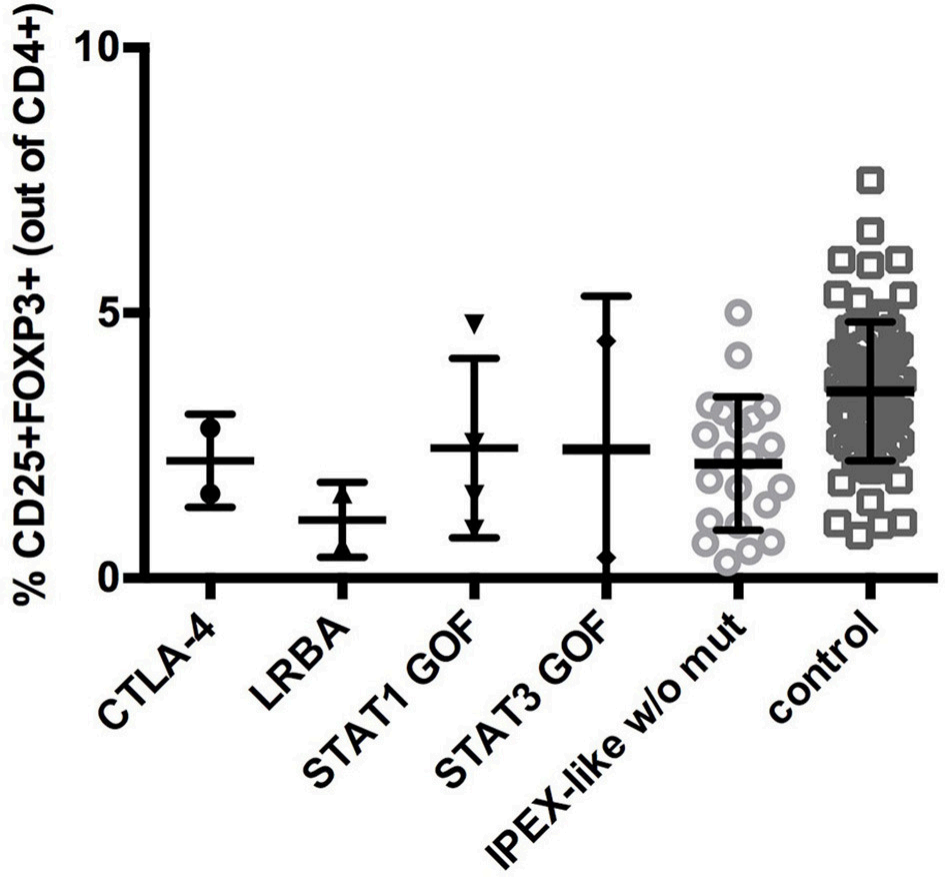
CD25+FOXP3+ expression in IPEX-like patients differed by the type of mutated genes identified and control subjects. Values are expressed as percentage of CD4 cells, mean and SD are shown.

In IPEX-like patients with mutations in *IL2RA* the percentage of CD4+FOXP3+ cells was within normal range, while CD25 expression, as expected, was absent, indicating a preservation of Treg quantity, but defective function (data not shown). Analysis of CD25 by flow cytometry in IPEX-like patients is essential to identify patients with CD25 deficiency. FOXP3+Tregs were evaluated in two of four patients with mutations in the *LRBA* gene. Both patients had a reduction in the percentage of Treg cells, in line with recently published data on patients with LRBA deficiency (35). In patients with CTLA4 deficiency the frequency of CD4^+^ Treg cells was normal or slightly decreased, suggesting that autoimmunity in these patients is caused by defective Treg function. Similarly, patients with *STAT1* GOF or *STAT3* GOF mutations had variable percentages of Treg cells, with a general tendency to decreased, but statistically not significant, values (Figure 4).

### Clinical phenotypes of IPEX and IPEX-like patients

**Figure 5:**
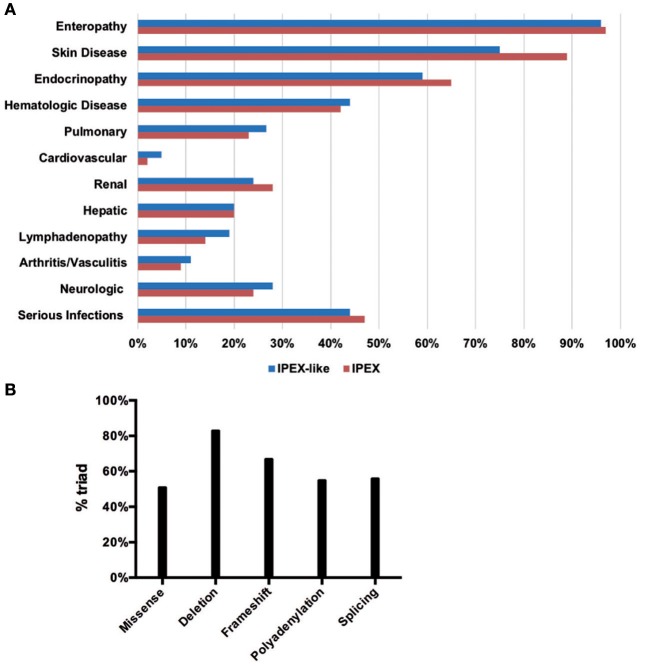
Clinical manifestations in the IPEX and IPEX-like cohorts **(A)**. IPEX patient stratified for type of mutation showing classical triad of symptoms **(B)**.

The clinical data obtained from IPEX and IPEX-like patients included in this study are summarized in Figures 5A,B and Supplementary Table 2. Enteropathy was the most consistent finding in both groups of patients (IPEX 97% vs. IPEX-like 96%, *p* = 1) and is most commonly associated with watery diarrhea, with a minority of patients reporting blood or mucus in the stools. Diarrhea begins within the first few months of life in the majority of patients in both groups.

For patients in whom age of onset was known, the mean age at onset of enteropathy among the IPEX patients was 7.8 months compared to 17.3 months in the IPEX-like patients (*p* = 0.15). Histologically, the incidence of villous atrophy on small bowel biopsy was similar (IPEX 45% vs. IPEX-like 34%, *p* = 0.16). Food allergies (which were defined as either an abnormal RAST IgE or clinical evidence of allergy to foods) were present more frequently among patients in the IPEX group (IPEX = 36% vs. IPEX-like 11%, *p* < 0.0001) and were reported as being “severe” in most cases. In one patient, the food allergies resolved after successful hematopoietic stem cell transplantation (HSCT). Not surprisingly, many patients from both groups suffered from failure to thrive (IPEX 75% vs. IPEX-like 71%, *p* = 0.61) which is likely due to persistent enteropathy, malabsorption, and chronic disease.

Skin manifestation were also widespread among both groups of patients (IPEX 89% vs. IPEX-like 75%, *p* = 0.03). There was however a greater difference in the incidence of eczema and/or exfoliative dermatitis (IPEX 85% vs. IPEX-like 65%, *p* = 0.003). Erythroderma (IPEX 8% vs. IPEX-like 11%, *p* = 0.6) and childhood alopecia (IPEX 11% vs. IPEX-like 15%, *p* = 0.5) were reported in both groups.

Autoimmune endocrinopathies, which most commonly include type I diabetes mellitus (T1DM) and hypo- or hyperthyroidism and, in a few patients, adrenal insufficiency, were seen at a similar frequency among the IPEX and IPEX-like patients (IPEX 65% vs. IPEX-like 59%, *p* = 0.44). However, there was almost a two-fold difference in the number of patients with T1DM in the IPEX cohort compared to the IPEX-like group (IPEX 49% vs. IPEX-like 28%, *p* = 0.008). For those with known age of onset of T1DM, IPEX patients tended to present earlier (mean = 27 months) than the IPEX-like patients (mean = 36 months) (*p* = 0.009). A few patients in both groups had positive Anti-glutamic acid decarboxylase (GAD) antibodies or Anti-islet cell antibodies in the absence of clinical disease (IPEX *n* = 5, IPEX-like *n* = 5). Interestingly, one carrier mother with a heterozygous mutation of *FOXP3*, had clinical evidence of T1DM.

Clinical thyroid disease (defined as having abnormal Thyroid Stimulating Hormone [TSH], triiodothyronine [T3], or free thyroxine [T4] with or without symptoms) was slightly more prevalent in the IPEX-like group (IPEX 26% vs. IPEX-like 39%, *p* = 0.10). A small number of patients in both groups had evidence of autoantibodies against thyroid (anti-microsomal or anti-thyroglobulin) but lacked evidence of clinical disease (IPEX *n* = 3, IPEX-like *n* = 5).

When evaluating patients for the presence of the classic triad of enteropathy, endocrinopathy, and skin manifestations, IPEX patients were more likely to be affected by all three (IPEX 58% vs. IPEX-like 44%, *p* = 0.07).

Of the other autoimmune phenomena typically associated with IPEX syndrome, hematologic abnormalities were equally present in both groups (IPEX 42% vs. IPEX-like 44%, *p* = 0.88), including anemia (IPEX 33% vs. IPEX-like 42%, *p* = 0.87), neutropenia (IPEX 13% vs. IPEX-like 15%, *p* = 0.66), and thrombocytopenia (IPEX 22% vs. IPEX-like 20%, *p* = 0.85). Organ and non-organ specific autoantibodies were also present in both groups (IPEX 17% vs. IPEX-like 26%, *p* = 0.26).

Renal abnormalities were common in both groups (IPEX 28% vs. IPEX-like 25%, *p* = 0.61) characterized by glomerulonephritis, interstitial nephritis, unexplained hypertension, persistent hematuria, or proteinuria. Hepatic abnormalities, most commonly autoimmune hepatitis, were found in both groups (IPEX 20% vs. IPEX-like 20%, *p* = 1). Pulmonary disease, such as asthma and interstitial lung disease (IPEX 23% vs. IPEX-like 27%, *p* = 0.60), lymphadenopathy (IPEX 14% vs. IPEX-like 19%, *p* = 0.41) were reported in both groups, although slightly more prevalent in the IPEX-like cohort. Arthritis or vasculitis (IPEX 9% vs. IPEX-like 11%, *p* = 0.80) were equally described. Few patients suffered from cardiovascular disease, mostly in the IPEX-like group (IPEX 2% vs. IPEX-like 5%, *p* = 0.44). This included pericarditis, atrial flutter, dilated aortic root, pericardial effusion, and aneurisms.

Approximately a quarter of patients in both groups were reported to have neurologic abnormalities (IPEX 24% vs. IPEX-like 28%, *p* = 0.60) including seizure disorders (IPEX 14% vs. IPEX-like 7%, *p* = 0.21), ventriculomegaly (IPEX 3% vs. IPEX-like 5%, *p* = 0.72), and developmental delay (IPEX 14% vs. IPEX-like 24%, *p* = 0.12), which, in our cohort, is higher than has been reported in the literature.

Serious infections were reported in almost half of all patients and included sepsis, meningitis, peritonitis, and pneumonia (IPEX 47% vs. IPEX-like 44%, *p* = 0.76). The most common organisms identified were Staphylococcus (IPEX 23% vs. IPEX-like 20%, *p* = 0.71), Cytomegalovirus (IPEX 10% vs. IPEX-like 7%, *p* = 0.59), and Candida (IPEX 19% vs. IPEX-like 15%, *p* = 0.55).

In order to assess a possible genotype-phenotype correlation within the IPEX cohort, we stratified IPEX patients for mutation type (frameshift/in frame deletion/missense/splice site/polyadenylation mutations) and correlated the genotype with the development of the classical clinical triad of immune dysregulation (Figure 5B). The complete phenotype was mainly present in patients affected by in-frame deletions (83%) and frameshift mutations (67%). In the other genotypes the clinical presentation was variable. Similarly, patients lacking exon 2 show a phenotype which do not significantly differ from other IPEX genotypes. However, the severity of symptoms is somehow unpredictable and not necessarily related to the mutation type.

**Figure 6:**
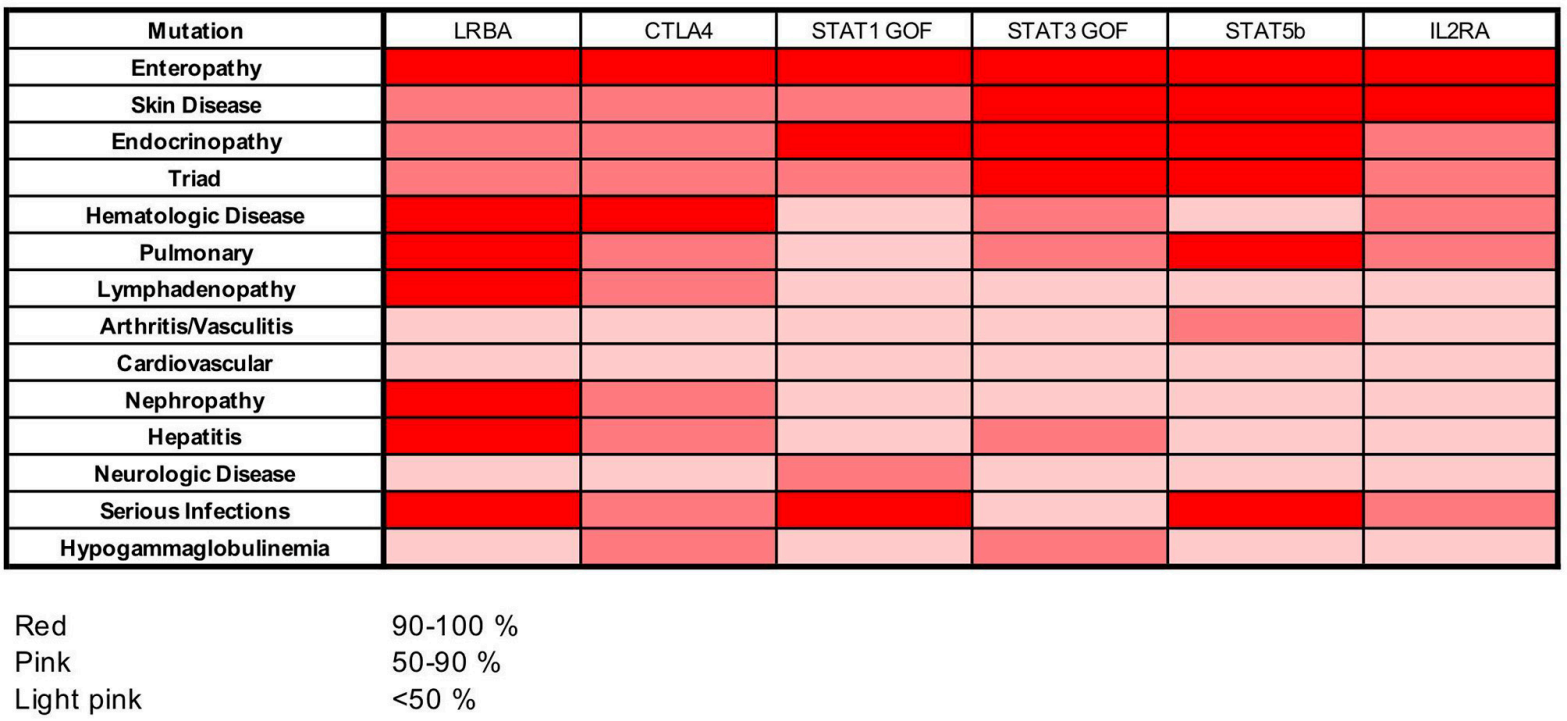
Heat map showing frequencies of clinical manifestations in our cohort of patients with *LRBA, CTLA4, STAT1 GOF, STAT3 GOF, STAT5b*, and *IL2RA* mutations.

Among the IPEX-like cohort, patients with mutations in genes related to Treg function (i.e., *CD25, STAT5b, STAT3* and *STAT1 GOF, LRBA, CTLA4*) are affected by a clinical phenotype strongly resembling IPEX. Most of them suffer from the classical clinical triad of immune dysregulation symptoms (enteropathy, skin disease, and endocrinopathy). All patients with heterozygous *CTLA4* mutations and patients with *LRBA* mutations suffer from autoimmune cytopenias. The majority of *LRBA* patients (75%) also developed lymphoadenopathy and/or hepatosplenomegaly, whereas serious viral and bacterial infections seem to cluster mainly in subjects with mutations in *CD25, STAT1GOF*, and *STAT5b* mutated subjects. Food allergies were not reported in this group (Figure 6).

### Laboratory findings

**Figure 7:**
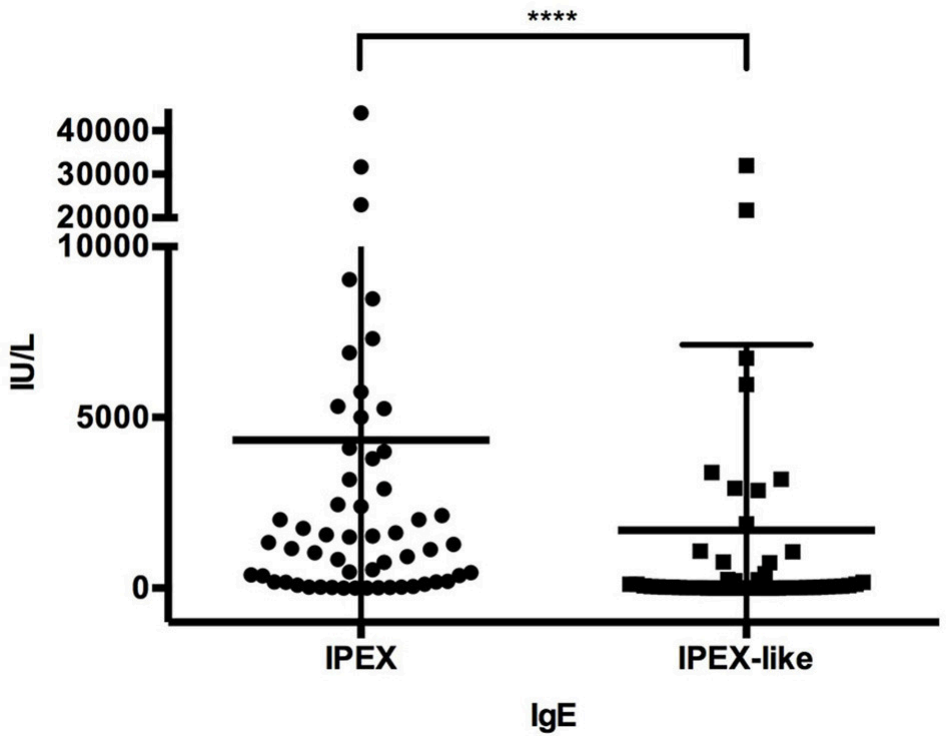
IgE levels in IPEX and IPEX-like patients. Mean and SD are shown (^****^p < 0.0001).

Immunoglobulin levels were generally normal in both groups of patients, except for elevated IgE levels in 92% of IPEX and 49% (*p* < 0.0001) of IPEX-like patients. IgE levels were >1,000 IU/ml in 59% of IPEX patients and 25.5% (*p* = 0.0005) of IPEX-like patients, some in each group having levels over 10,000 IU/ml (Figure 7). Hypogammaglobulinemia, if present, was associated mostly with *LRBA, STAT3 GOF*, and heterozygous *CTLA4* mutations. Absolute lymphocyte counts and CD4^+^ and CD8^+^ lymphocyte subsets were normal in most IPEX and IPEX-like patients. *In vitro* lymphocyte proliferation in response to mitogens and antigens were also normal in most patients as well as antibody responses to immunization with protein antigens (data not shown).

### Treatment

Most IPEX patients responded at least temporarily to the combination of steroids with either cyclosporine A, tacrolimus, or sirolimus. However, many patients in both groups had only marginal or poor responses to therapy with steroids alone. Hematopoietic stem cell transplantation (HSCT) was performed in 40 IPEX patients and 15 IPEX-like patients. Nine patients with IPEX (22.5%) but only one with IPEX-like syndrome died post-transplantation from transplant- and disease-related complications. Most surviving patients had resolution of their enteropathy and skin disease post-transplantation, whereas T1DM, when present at the time of transplantation, persisted.

### Prognosis

Time (in years) of follow-up since birth was obtained on 163 out of 173 patients.

**Figure 8:**
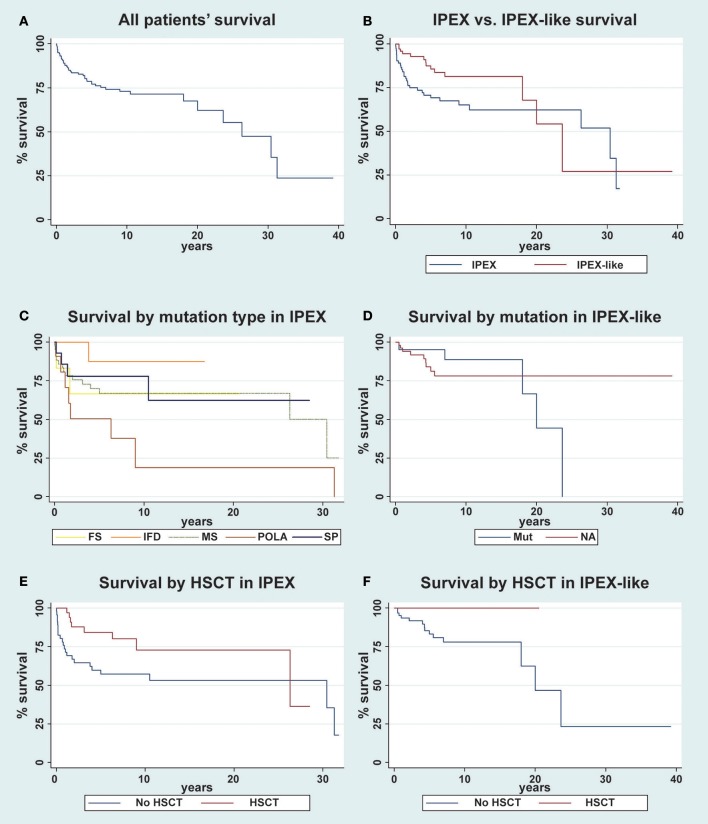
Kaplan-Meier survival curve for overall survival of patients. **(A)** The overall survival in the whole cohort of patient is evaluated (*n* = 163); **(B)** IPEX (*n* = 85) vs. IPEX-like (*n* = 78) patients are compared; **(C)** IPEX patients stratified for type of mutation: frameshift (FS) *n* = 6, in frame deletion (IFD) *n* = 11, missense (MS) *n* = 43, splicing mutations (SP) *n* = 14 and mutations in polyadenylation site (POLA) *n* = 11 are compared; **(D)** IPEX-like without mutations (NA= not available) *n* = 57 and IPEX-like with mutation (Mut) *n* = 21 are compared; **(E)** IPEX patient stratified whether (*n* = 39) or not (*n* = 46) they underwent HSCT and **(F)** IPEX-like patient stratified whether (*n* = 10) or not (*n* = 68) they underwent HSCT.

Overall, long term survival (30 years) of all patients evaluated (*n* = 163) was 47.5% (Figure 8A). When comparing survival data between IPEX and IPEX-like patients, we observed that long term survival (30 years) was poor in both groups of patients with a better survival rate in the IPEX group (52% IPEX vs. 27% IPEX-like), but with a greater 10-year survival in the IPEX-like group (81.5%) compared with IPEX patients (65%) (Figure 8B). When stratified by the type of mutation, we observed the highest 10-year survival in patients with in frame deletions (87.5%), followed by splice site mutations (78%), missense mutations (67%), frameshift (67%), and polyadenylation site mutations (19%) (Figure 8C).

When stratifying the IPEX-like cohort into two groups, subjects with a single gene defect (IPEX-like with mutation) and those without an identified genetic defect (IPEX-like without mutation) and then comparing survival data in these two IPEX-like groups with IPEX patients, we observe a higher long-term survival (20 years) in IPEX-like without mutation (78.3%) compare with IPEX patients (62.4%) and with IPEX-like with mutations (44.4%) (Figure 8D). IPEX patients showed the worst early survival (10-year survival) when compared with the other groups.

Finally, we evaluate the effect of HSCT both in the IPEX and IPEX-like groups. We observed a 10-year survival of 72.8% in IPEX patients who underwent HSCT (*n* = 38) and 57.3% in non-transplanted (*n* = 46) IPEX patients (*p* = 0.02) (Figure 8E). After 15 years, several transplanted IPEX patients were lost to follow-up causing the drop in survival curve. In the IPEX-like cohort we observed a 10-year survival of 100% in transplanted patients (*n* = 10) and 78% in non-transplanted (*n* = 68) patients (*p* < 0.0001) (Figure 8F).

## Discussion

IPEX is a syndrome of systemic autoimmunity caused by mutations in *FOXP3*, which codes for a member of the forkhead transcription factor family, expressed primarily in CD4^+^CD25^+^ regulatory T cells. A similar syndrome is caused by the naturally occurring *Foxp3* mutation in mice resulting in the *scurfy* phenotype characterized by diarrhea, runting, a scaly skin rash, anemia, thrombocytopenia, lymphadenopathy, hepatosplenomegaly, and early death. To define the clinical phenotype associated with mutations in *FOXP3*, we collected data from 173 patients from 143 unrelated families with phenotypic features of IPEX, who were referred to us for mutation analysis and of which 88 had mutations in *FOXP3*.

The 44 unique *FOXP3* mutations identified in our cohort of IPEX patients clustered in the functional domains, particularly in the leucine zipper and forkhead (FKH) domains. Residues located in the FKH domain play a role in the interaction of FOXP3 with NFAT, NF-KappaB, DNA binding, and the formation of domain swapped dimers (101–103). Several “hot-spot” mutations were identified in *FOXP3* with four of these mutations accounting for IPEX in almost 1/3 of the families: two of these hot-spot mutations were single amino acid deletions located in the leucine zipper region (p.K250del and p.E251del) found in 8 patients, and two were missense mutations in the FKH domain, p.R337Q and p.A384T, affecting one of the DNA binding site residues, observed in 12 patients. The p.A384T mutation is commonly associated with a mild phenotype and has recently been shown to impair the suppressive function of Treg cells while maintaining most aspects of FOXP3 transcriptional functions including the ability to repress inflammatory cytokines. The impaired suppressive function of Tregs with the p.A384T substitution is in part due to a specific disruption of the binding of the mutated FOXP3 to the histone acetyltranferase Tat-interacting protein 60 (TIP60); this defect can be corrected using allosteric modifiers that enhance FOXP3-TIP60 interaction (104).

FOXP3 expression varied in IPEX patients depending on the type of mutation with low or lack of expression associated with in-frame and frameshift deletion mutations, low expression in polyadenylation site and splicing mutations, and variable expression in patients with missense mutations. IPEX-like patients were found to have generally low levels of FOXP3 expression compared to controls, in particular those affected by mutations in genes related to Treg function (i.e., *LRBA, CTLA4*). This finding is expected since the *scurfy*/IPEX phenotype is explained by extensive immune dysregulation resulting from the absence or abnormal function of CD4^+^CD25^+^ regulatory T lymphocytes.

Of the 143 families (173 patients) analyzed, 63 families (77 patients) had mutations within the coding region of *FOXP3* or exon-intron junctions. Three families with multiple affected members were identified to have *FOXP3* mutations in the canonical Poly-A signal 878–883 bp downstream of the termination codon. Two families had the AAUAAA > AAUGAA mutation originally described by Bennett et al. (42), one of which is the family reported by Powell et al. (2). Affected members of the other family carried the AAUAAA > AAUAAG mutation. Additional reports of Poly-A mutations causing IPEX suggest an unusually high incidence of mutations in the Poly-A site of FOXP3 (60, 61, 77). In the PID field, only 2 cases of X-linked SCID due to Poly-A mutations (AAUAAA > AAUAAG) in the IL-2Rgamma chain were reported [(105) and Walter J, unpublished]. Mutations affecting the canonical Poly-A site are rare, except in patients with alpha Thalassemia due to Poly-A mutations in the *alpha-globulin* gene (106, 107), or in patients with beta-Thalassemia due to Poly-A mutations in the *beta-globulin* gene (108, 109). The remaining 77 families (85 patients) had a similar phenotype but without demonstrable mutations in the coding region, the upstream non-coding region, or the polyadenylation site of *FOXP3*. These patients were termed “IPEX-like” patients. The clinical findings of these two groups of patients are summarized in Figure 5 and Supplementary Table 2. IPEX patients more likely had food allergies, eczema, T1DM, as well as the full triad of classical symptoms (enteropathy, skin disease, and endocrinopathy), while IPEX-like patients seemed to be more frequently affected by thyroid disease and cardiovascular abnormalities.

Much of what is known about the clinical characteristics of IPEX syndrome is derived from case reports (2–8, 42, 45, 52–54, 57, 58, 66–68, 70, 71, 73) or reviews (110, 111). Compared to the literature we found in our IPEX cohort a higher frequency of characteristics that had been associated with this syndrome, likely the result of a more comprehensive data collection. Some features were strikingly frequent in the IPEX group, including failure to thrive, food allergies, alopecia, and the presence of the complete “classic triad.” Neurologic abnormalities were present in 28% of our patients including seizures, developmental delay or ventriculomegaly. The incidence of neurologic abnormalities was almost 3 times as high as reported by Barzaghi et al. (111). Central nervous involvement may represent an autoimmune phenomenon, however we presume to be probably caused by chronic disease, metabolic derangements and immunosuppressive therapy, especially since IPEX-like patients seem to be equally affected. Similarly, the severe infections that are quite common in both groups, considering the pathogens involved (i.e., *Staphylococcus, Candida albicans, Cytomegalovirus*), are most likely a complication of intense and prolonged immune suppression rather than a consequence of an imbalanced immune system.

While elevated in both groups, IPEX patients had significantly higher levels of IgE compared to IPEX-like patients, possibly a useful diagnostic tool.

The long-term (30 years) survival of both groups is poor (52% IPEX vs. 27% IPEX-like). However, the 10-years survival of 81.5% in the IPEX-like group, particularly in those patients not genetically defined, is significantly higher when compared with the IPEX cohort (65%). This suggests that while there is a difference in the survival curves, depending on the presence and type of the *FOXP3* mutation or the presence of a mutation in related genes, the long-term consequences of chronic autoimmunity and immunosuppressive therapy and resulting infections are affecting outcome and patients' quality of life. In both, the IPEX and IPEX-like cohorts, HSCT improves the survival rate, supporting early HSCT as curative treatment (Figures 8E,F).

The progress of DNA sequencing technologies has rapidly expanded the group of IPEX-like disorders caused by mutations in genes coding for proteins involved primarily (i.e., IL2Ra, STAT5b, CTLA4) and secondarily (i.e., LRBA, STAT3-GOF, STAT1-GOF) in Treg generation and function, by playing important roles in maintaining immune homeostasis (112). However, the phenotypes of these genetically diverse entities are often overlapping and a definite diagnosis requires sophisticated molecular and functional analyses.

We were surprised to identify several genes in our IPEX-like cohort that were not directly linked to immune regulation. Mutations in *TTC7A* (83, 113) and *TTC37* (100, 114) have been associated with severe diarrhea, villous atrophy, recurrent infections and (in TTC7A) a SCID-like phenotype with multiple intestinal atresia. DOCK8 deficiency shares with IPEX some features such as atopic dermatitis, autoimmunity, recurrent infections including muco-cutaneous candidiasis but, with few exceptions (99), not inflammatory bowel disease. While the clinical phenotypes of these conditions are generally quite dissimilar from IPEX and IPEX-like disorders, they should be considered in the differential diagnosis.

**Figure 9:**
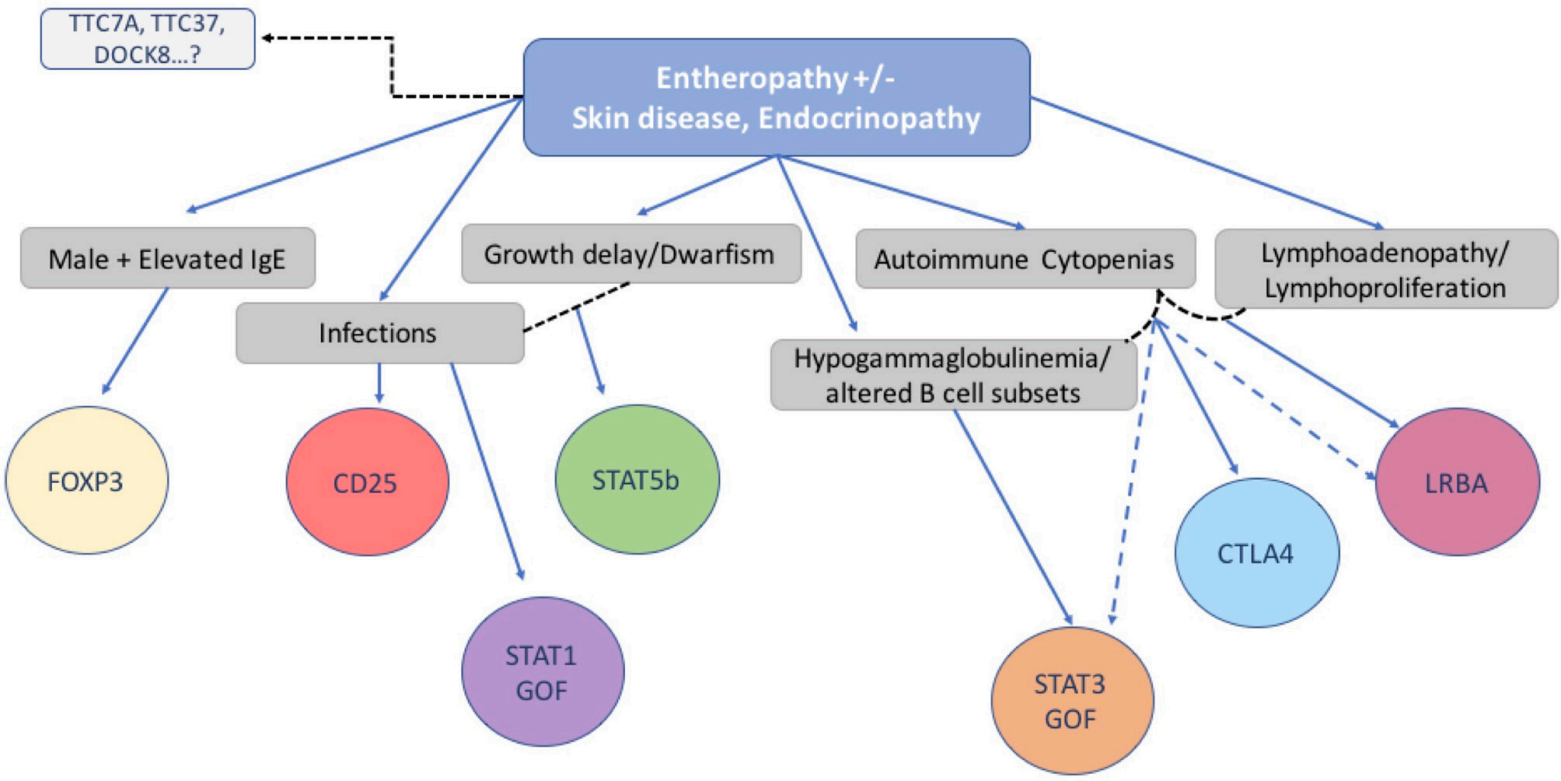
Flow chart suggesting molecular analysis strategy based on patients' clinical phenotype and immunological markers.

In conclusion, we have investigated the clinical features, immunologic characteristics and molecular basis of 173 consecutive patients with a phenotype compatible with IPEX. While half of the group had IPEX caused by a pathogenic mutation in the *FOXP3* gene, the other half did not. In the IPEX cohort, we were able to link certain clinical features with mutation type and location of the variant in the *FOXP3* gene. To identify a possible genetic cause, we used a multiplex platform and Sanger sequencing of candidate genes which revealed a single gene defect in 27% of our cohort of 85 IPEX-like patients, involving 9 different genes, allowing genotype-phenotype comparisons. While we did not find unambiguous differences between IPEX and IPEX-like (with or without a mutation) patients, we nevertheless attempted to design a simple flow chart to assist in selecting an efficient diagnostic strategy (Figure 9). However, with the increasing number of relevant gene defects and the realization that atypical presentations are the norm, single gene sequencing is increasingly replaced by Next Generation Sequencing that includes designer multiplex platforms and Whole Exome Sequencing.

## Ethics statement

This retrospective chart review study was carried out in accordance with the Declaration of Helsinki and the recommendations of the Anna Meyer Children's Hospital Pediatric Ethics Committee, and Seattle Children's Hospital Institutional Review Board. It was deemed exempt but some patients also provided written informed consent for inclusion in associated clinical studies.

## Author contributions

EG, HO, and TT devised the project, wrote the manuscript, supervised the molecular analysis. SC carried out experiments, performed data analysis, wrote the manuscript. DH performed genetic and data analysis. MV carried out experiments, wrote the manuscript. SA-S supervised work in the laboratory and SR carried out experiments. GS and EA contributed with data collections and analysis. CF supervised the work.

### Conflict of interest statement

The authors declare that the research was conducted in the absence of any commercial or financial relationships that could be construed as a potential conflict of interest.

## Abbreviations

IPEX, Immune dysregulation, Polyendocrinopathy, Enteropathy: X-linked
PID: Primary Immune Deficiency
FOXP3: Forkhead box P3
T1DM: Type 1 diabetes mellitus
HSCT: Hematopoietic Stem Cell Transplantation
Treg: Regulatory T cells.
