# Supplementary material for: Clinical, Immunological, and Molecular Heterogeneity of 173 Patients With the Phenotype of Immune Dysregulation, Polyendocrinopathy, Enteropathy, X-Linked (IPEX) Syndrome
Source: Front Immunol. 2018 Nov 1;9:2411. doi: 10.3389/fimmu.2018.02411 (PMC6223101; doi:10.3389/fimmu.2018.02411)
Supplement: Supplementary file 1 [file Data_Sheet_1.PDF]

Supplementary Material

Clinical, Immunological and molecular heterogeneity of 173 patients with the phenotype of Immune Dysregulation, Polyendocrinopathy, Enteropathy, X-linked (IPEX) Syndrome

*Gambineri E.[1, 2], Ciullini Mannurita S.[1,2], Hagin D. [3], Vignoli M.[1,2], Anover-Sombke S.[3], Rylaarsdam S.[3], Segundo G.R.S. [3], Allenspach E.J. [3], Favre C.[2], Ochs H.D.[3], *Torgerson T.R.[3]

*** Correspondence:**
Corresponding Author: Eleonora Gambineri,
e-mail: eleonora.gambineri@unifi.it; e.gambineri@meyer.it
Troy R. Torgerson, MD PhD, Associate Professor of Pediatrics
e-mail: troy.torgerson@seattlechildrens.org

1 Supplementary Tables

Supplementary Table 1. List of the 50 genes included in the custom sequencing array we designed for NGS analysis.

GENE	GenBank N° (GRCh38.p7/hg38)
AIRE	NM_000383.3
CASP10	NG_007265.1, NM_032977.3
CASP8	NM_001228.4
CARD11	NM_032415.5
CD27	NM_001242.4
CORO1A	NM_007074.3
CTLA4	NM_005214.4
CTPS1	NM_001905.3
CTPS2	NM_019857.4
CXCR4	NM_003467.2
DOCK2	NM_004946.2
DOCK8	NM_203447.3
FAS	NM_000043.5
FASLG	NM_000639.2
FCGR3A	NM_001127593.1
FOXP3	NM_014009.3
GATA2	NM_032638.4
IKBKG (NEMO)	NM_001099856.4
IL10	NM_000572.2
IL10RA	NM_001558.3
IL10RB	NM_000628.4
IL2RA	NM_000417.2
ITCH	NM_001257137.2
ITK	NM_005546.3
JAK3	NM_000215.3
LRBA	NM_006726.4
MAGT1	NM_032121.5
MALT1	NM_006785.3
PGM3	NM_001199917.1
PIK3CD	NM_005026.3
PIK3R1	NM_181523.2
PIK3R5	NM_014308.3
PLCG2	NM_002661.4
PRKCD	NM_006254.3
RAG1	NM_000448.2

RAG2	NM_000536.3
SH2D1A	NM_002351.4
STAT1	NM_007315.3
STAT3	NM_139276.2
STAT4	NM_003151.3
STAT5A	NM_003152.3
STAT5B	NM_012448.3
STIM1	NM_003156.3
STK4	NM_006282.3
STXBP2	NM_006949.2
STX11	NM_003764.3
TMEM173(STING)	NM_198282.3
UNC13D	NM_199242.2
XIAP	NM_001167.3
WAS	NM_000377.2

Supplementary Table 2. Clinical manifestation in IPEX and IPEX-like cohort of patients. P<0.05 is considered as statistically significant.

Clinical Characteristics	IPEX	IPEXlike	p value
Enteropathy	97%	96%	1.000
Villous Atrophy	45%	34%	0.162
Failure to thrive	75%	71%	0.609
Food Allergies	36%	11%	< 0.0001
Skin Disease	89%	75%	0.029
Eczema	85%	65%	0.003
Erythroderma	8%	11%	0.607
Alopecia	11%	15%	0.506
Endocrinopathy	65%	59%	0.438
Diabetes Mellitus	49%	28%	0.008
Thyroid Disease	26%	39%	0.104
Classic Triad	58%	44%	0.069
Hematologic Disease	42%	44%	0.879
Anemia	33%	42%	0.873
Thrombocytopenia	22%	20%	0.853
Neutropenia	13%	15%	0.663
Pulmonary	23%	27%	0.598
Cardiovascular	2%	5%	0.438
Renal	28%	25%	0.609
Hepatic	20%	20%	1.000
Lymphadenopathy	14%	19%	0.412
Arthritis/Vasculitis	9%	11%	0.802
Neurologic	24%	28%	0.604
Seizures	14%	7%	0.214
Developmental Delay	14%	24%	0.118
Ventriculomegaly	3%	5%	0.717
Serious Infections	47%	44%	0.760
Staph	23%	20%	0.713
CMV	10%	7%	0.591
Candida	19%	15%	0.550
